# TRIP 13-dependent pathways promote the development of gastric cancer

**DOI:** 10.1007/s10142-023-01160-7

**Published:** 2023-07-11

**Authors:** Fengming Ni, Xinmin Liu, Yan Xia, He Zhu, Fudong Li, Nan Zhang, Hong Xu

**Affiliations:** 1 Department of Gastroenterology, The First Hospital of Jilin University, No. 1 Xinmin Street, Changchun, 130021 China; 2 Department of Neurology, The First Hospital of Jilin University, Changchun, China

**Keywords:** TRIP13, Gastric cancer, Oncogenic pathways, Survival relevance, Gene set enrichment analysis (GSEA)

## Abstract

TRIP13 is highly expressed in various human tumors and promotes tumorigenesis. We aimed to explore the biological effect of TRIP13 on gastric cancer. The RNA sequence data were retrieved from TCGA to evaluate TRIP13 mRNA expression in gastric cancer. Paired formalin-fixed paraffin-embedded blocks were further analyzed to verify the relationship between TRIP13 expression and carcinogenic status. The functions of TRIP13 on the proliferation of gastric malignancy were investigated by MTT, flow cytometry, colony formation experiment, and nude mouse tumor formation experiment. Finally, microarray analysis of TRIP13-related pathways was performed to identify the potential underlying mechanism of TRIP13 in gastric cancer. TRIP13 was found to have high expression in tumor samples. TRIP13 expression status was significantly subjective to tumor-node-metastasis (TNM) staging and poor survival. The downregulation of TRIP13 promoted apoptosis and inhibited tumor growth. TRIP13-dependent JAK/STAT and NF-κB signaling cascade were found as two key pathways in the carcinogenesis of GC. In conclusion, TRIP13 participates in the carcinogenesis of stomach cancer, and its overexpression in the cancerous tissues dovetail with advanced stage and survival. Moreover, TRIP13 functions as an upstream regulator of the JAK/STAT and p53 signaling pathways, which play critical roles in developing various malignancies.

**Supplementary Information:**

The online version contains supplementary material available at 10.1007/s10142-023-01160-7.

## Introduction

Gastric cancer is a prevalent malignant disease entity, accounting for 5.7% of new cancer cases each year, with mortality following only lung and colorectal cancers (Bray et al. 2018). Recent epidemiological studies have shown decreased incidence and mortality of gastric malignancy worldwide (Luo et al. 2017), but the yearly cost of its treatment still remains high because of the lack of effective therapy (Thrift and El-Serag 2020). Therefore, an urgent search for potential therapeutic targets for gastric cancer has been called. After sorting and analyzing The Cancer Genome Atlas (TCGA) database in our first-phase research, thyroid hormone receptor interactor 13 (TRIP13) was singled out as a candidate for the targeted treatment of gastric malignancy.

Thyroid hormone receptor–interacting factor 13 (TRIP13) exhibits conserved expression in many species, since it is an ATPase family member and functions in a variety of cellular-active protein families. TRIP13 was first discovered in yeast, where a lack of TRIP13 leads to cell cycle arrest (San-Segundo and Roeder 1999). TRIP13 forms a stable hexameric ring, and ATP binding, as well as ATP hydrolysis, is critical for the function of the protein. Subsequent studies found the human *trip13* gene on chromosome 5 was translated into a functional protein with 432 amino acid residues, which was categorized as to the large AAA + protein superfamily of ring-shaped P-loop NTPases, and involved in checkpoint regulation, double-strand DNA break repair, and chromosome synapsis formation during meiosis and so on (Bhalla and Dernburg 2005; Joyce and McKim 2009, 2010). Despite recent studies on TRIP13 in the human head and neck (Banerjee et al. 2014), prostate (Larkin et al. 2012), and colorectal cancers (Sheng et al. 2018) have suggested its involvement in drug resistance and carcinogenesis, the oncogenic function of TRIP13 in gastric malignancy remains largely unknown.

In the initial phase of our study, TCGA database was screened to compare the divergence in the TRIP13 expression in both normal and cancerous gastric tissues and confirmed that the high level of TRIP13 expression indeed predicts poor survival in GC patients. Herein, in order to further investigate the carcinogenic role of TRIP13 in gastric malignancy, we proceeded with experiments and bioinformatic analyses to explore potential downstream genes of TRIP13 and preliminarily demonstrated the carcinogenic functions of TRIP13 in gastric malignant cells. Taken together, these data demonstrated that TRIP13 promoted gastric cell tumorigenesis through the JAK/STAT and p53 signaling pathways, and they identify a promising therapeutic target in the treatment of gastric cancer.

## Materials and methods

### Data resource

The sequence data of RNA from 375 neoplastic tissues and 32 non-cancerous tissues of the stomach were retrieved from TCGA to evaluate TRIP13 mRNA expression. Twenty-seven paired samples were further analyzed to verify the relationship between TRIP13 expression and carcinogenic status. The obtained bioinformatic data were then processed by R 3.6.3 for detailed analysis.

### Tissue samples with the clinicopathological information

The cancerous and paired normal tissues from 98 gastric cancer patients were obtained from a biotech company (Outdo, China). All samples were stored in a − 80 °C freezer for further protein extraction. Formalin-fixed paraffin-embedded blocks were created using the samples from 98 GC patients between July 2006 and April 2007. These patients were followed up every 6 months with a complete check-up until September 2014.

### Immunohistochemical analyses

All formalin-fixed paraffin-embedded blocks were sliced into 5 μm sections for microscopic examination. Then, the slides with the mounted sections were heated at 60℃ for 40 min, and then deparaffinized as well as rehydrated according to the protocol by Tan et al. (2015) After incubating the slides in 10 nM citrate antigen retrieval solution (Abcam, Cambridge, MA, USA) at 95℃ for 10 min, and then blocking in 3% goat serum to prevent non-specific binding, polyclonal antibodies against TRIP13 (Abcam) and Ki-67 (Proteintech, Chicago, IL, USA) were sequentially added to the slides at a concentration of 1:150 and 1:400, respectively, following overnight incubation at 4℃, five fields were randomly chosen in each slide and microscopically examined by two pathologists independently.

### Cell culture

The human gastric malignant cell lines SGC-7901, AGS, MGC-803, BGC-823, and MKN-45 were obtained from Shanghai Genechem (Shanghai, China), and MGC-803 and AGS, which have been extensively studied. All cells were placed in Dulbecco’s modified Eagle medium with 10% fetal bovine serum. Penicillin (100 U/ml) and streptomycin (100 U/ml) were added to the medium to prevent contamination. Cells were incubated at 37 °C in a humidified atmosphere containing 95% air and 5% CO2.

### Cell transfection with lentiviral vector harboring human TRIP13 cDNA

The TRIP13 cDNA from PCR amplification was subcloned into the pGV112 plasmid. For the knockdown of TRIP13, two human TRIP13-targeting small interfering RNA (siRNA) sequences were cloned into lentiviral vectors. For vial transduction, cells in the exponential growth phase were treated with trypsin, and subsequently re-cultured in the medium at a concentration of 3–5 × 10^4^ cells/ml. After 72 h of infection, the fluorescence and infection efficiency were determined using an inverted fluorescence microscope at 200 × magnification (IX-71; Olympus Corporation). The medium was replaced and the optimal number of lentiviruses with siRNA was added. At 8–12 h following infection, the lentivirus-containing medium was changed and the lentivirus-transfected cells were cultivated for future use.

### Western blotting

Western blotting was performed upon standard protocol (Sheng et al. 2018). The primary antibodies, including anti-interleukin 6 (IL-6), anti-caspase-3, and anti-TRIP13, were purchased from Santa Cruz, USA. Protein concentrations of cell lysates were determined using the Bradford method. Equal amounts of protein (20 µg/lane) were separated using 12% SDS-PAGE and transferred to a PVDF membrane. The membranes were blocked using 5% BSA, at 25 °C for 2 h. The membrane was further incubated with primary antibodies and then second antibodies. Membranes were re-washed three times with TBST buffer and finally visualized with enhanced chemiluminescence (Millipore, USA) and the ChemiDoc MP System (Bio-Rad, USA).

### Gene set enrichment analysis

In order to explore the TRIP13-related contribution to a poor prognosis in gastric malignancy, gene set enrichment analysis (GSEA) (Ni et al. 2022; Xu et al. 2022) was performed to determine whether various known genes showed a significant difference between groups with high and low TRIP13 expression (Mootha et al. 2003; Subramanian et al. 2005). The present study designated the gene set with *P* < 0.05 and false discovery rate (FDR) < 0.25 as the significant enrichment.

### Cell viability assays

An MTT assay (Sigma-Aldrich; Merck KGaA) was performed to assess cell proliferation following after knockdown of TRIP13 as previously described. Briefly, lentivirus-infected cells were seeded in 96-well plates at 3000 cells/well. At 1, 2, 3, 4, and 5 days following incubation, 20 µl MTT solution (1 mg/ml) was added to each well and incubated at 37 °C for 4 h. Subsequently, the medium was carefully removed and 100 µl acidic isopropanol (10% SDS, 5% isopropanol, and 0.01 mol/l HCl) was added to each well. The plates were read using an automated microplate reader (Molecular Devices, LLC) at 490 nm.

### Colony formation assay

shTRIP13-infected and shCtrl cells were digested with trypsin and resuspended in a standard medium after reaching the logarithmic growth phase. Cells were seeded into 6-well plates at a density of 500 cells/well, incubated at 37 °C in a 5% CO_2_ incubator, and observed for 10 days with half of the medium changed every 3 days. Cells were washed with PBS and fixed with 4% paraformaldehyde (1 ml/well; Shanghai Sangong Pharmaceutical Co., Ltd.) for 30–60 min at room temperature. Cells were washed with PBS, stained with 500 µl Giemsa (cat. no. ECM550; Chemicon; Thermo Fisher Scientific, Inc.) for 20 min at room temperature, and washed with deionized water three times. Images of cell colonies were captured using an inverted light microscope (200 × magnification; IX71; Olympus, Tokyo, Japan) and counted using ImageJ software (version 4.0; National Institutes of Health).

### Flow cytometry

Apoptosis assessment was performed using an Annexin-V/FITC Apoptosis Detection kit (Invitrogen; Thermo Fisher Scientific, Inc.) according to the manufacturer’s protocols. Briefly, cells were infected with shTRIP13 or shCtrl. Cells were harvested and stained with Annexin-V/FITC and propidium iodide (PI) in a binding buffer for 15 min at room temperature in the dark. The samples were analyzed using a BD FACScan system (BD Biosciences) to determine the percentage of cells exhibiting Annexin-V and PI staining. Apoptotic cells were subsequently analyzed via flow cytometry, using MoFlo XDP (Beckman Coulter, Inc.).

### Tumor formation in nude mice

The MGC-803 human GC cell line was first transduced with lentiviruses harboring either LVpGCSIL-004PSC24145-1 or PSC3741 control, and then 5 × 10^6^ transduced cells were implanted subcutaneously at the right flank of 4-week-old BALB/c nude mice. Dimensions of the newly formed tumors were recorded per 3 days for calculating the tumor volume by the equation of (length × width^2^)/2 starting from 1 week after the injection. Following 3 weeks of breeding, the mice underwent sacrificing in order to harvest the formed tumor.

### Statistical analyses

The differences between different groups were compared either with a two-tailed *t*-test or *χ*^2^ test. The effects of different variables on patient survival were investigated by Cox proportional hazard regression, Kaplan–Meier survival analysis coupled with log-rank test was attempted to compute gastric cancer patient overall survival, and Spearman’s correlation was performed for exploring correlation. All data were presented as the mean ± standard deviation, and *p* < 0.05 was designated as the statistical significance. All cut-off values for separating high and low expression groups were determined by the online R2 database algorithm. In the in vivo tumor formation experiment, each group had 10 replicates. All other experiments were conducted with 3 replicates in each group.

## Results

### TRIP13 increase as the poor prognostic sign of gastric malignancy

**Fig. 1:**
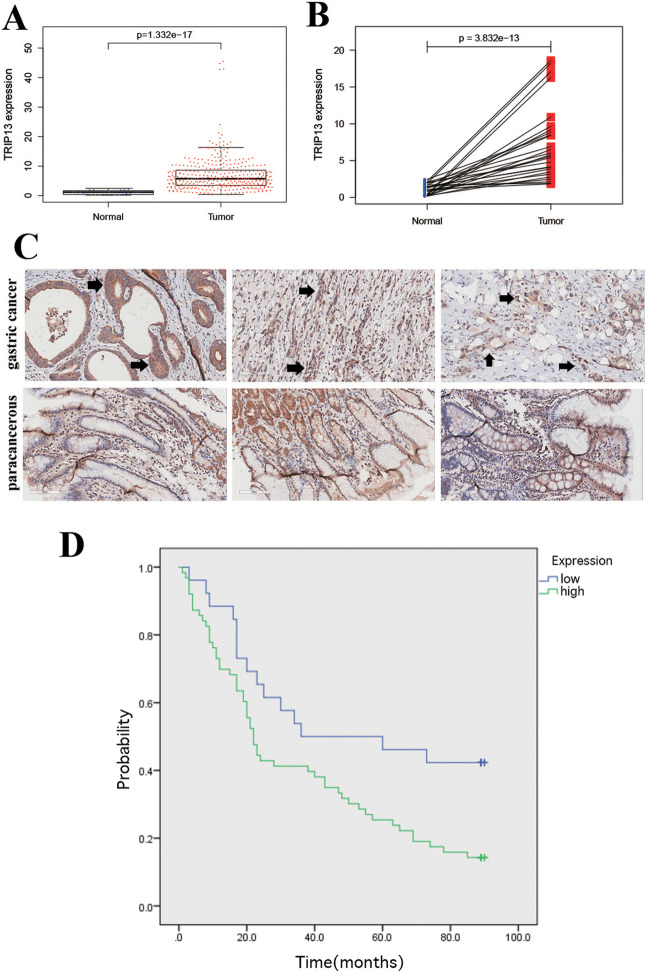
Overexpression of TRIP13 in gastric malignancy and its association with the inferior prognosis. **A** TCGA dataset showing TRIP13 upregulation in gastric cancer tissues *(P* < 0.01). **B** TRIP13 possessed overtranscription in GC tissues in 27 paired tissues from TCGA (*P* < 0.01. **C** Expression of TRIP13 in normal and malignant gastric tissues through immunohistochemistry. **D** Kaplan–Meier analysis of progression-free survival data with the log-rank test *P*-values indicated

**Fig. 2:**
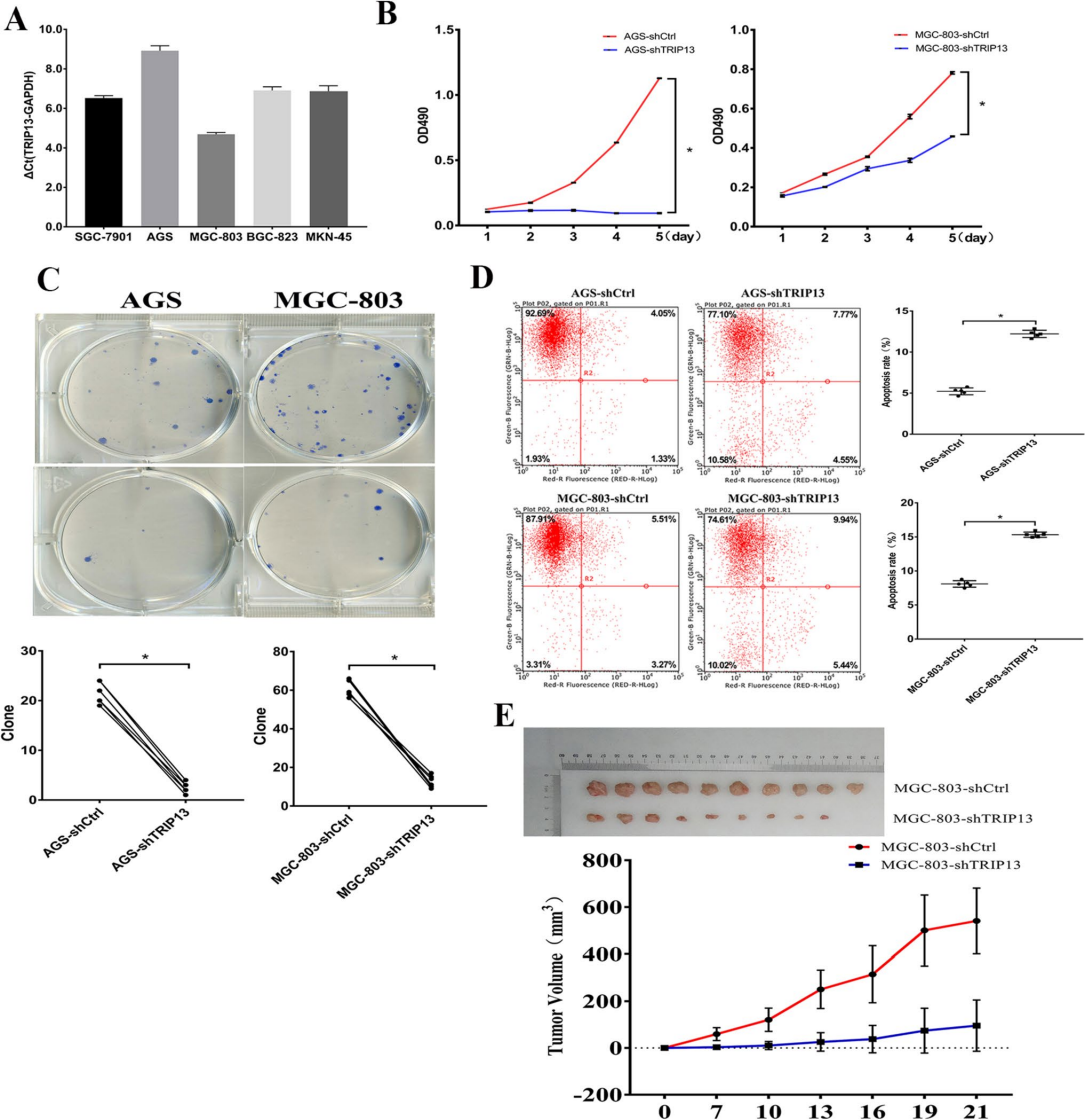
Altered TRIP13 in the cells from stomach cancer and its in vitro and in vivo effect on tumorigenic properties. **A** Relative RNA levels were determined by qPCR, when ΔCt ≤ 12, the expression of TRIP13 was abundant. **B** Result from TRIP13 inhibition on the proliferation of AGS and MGC-803 cells. **C** Result from TRIP13 inhibition on cell clone formation ability of AGS and MGC-803 cells. **D** Result of TRIP13 inhibition on the apoptosis of AGS and MGC-803 cells. **E** Images and statistical analyses of subcutaneous tumors formed by LVpGCSIL-shTRIP13-infected MGC-803 cells and psc3741-shCtrl-infected MGC-803 cells

By analyzing gastric malignancy and para-neoplastic samples in TCGA database, TRIP13 was found to have high expression in these samples (Fig. 1A; *P* < 0.001). The increase in expression of TRIP13 in the malignant cells of the stomach was further confirmed by comparing 27 paired cancer and para-neoplastic samples (Fig. 2A; *P* < 0.001). Immunohistochemical analyses of malignant and adjacent normal samples in the cancerous stomach also showed overexpression of TRIP13 in gastric cancer cells (Fig. 1C). The prognosis of the patients with gastric malignancy was further analyzed to show that the survival rate of the TRIP13 overexpression group was worse than that of the under-expression group (hazard ratio [HR]: 1.25, *P* < 0.05; Fig. 1D). Cox proportional hazard regression (Table 1) confirmed TRIP13 as an independent prognostic factor to predict survival possibility in the patients with gastric malignancy (HR = 0.502, *P* < 0.05).

**Table 1:** Univariate and multivariate analyses of the factors correlated with the overall survival of GC patients

Variable	Univariate analysis			Multivariate analysis		
	HR	95% CI	*P* value	HR	95% CI	*P* value
Expression	.704	1.137–3.592	.016*	.502	.272–.924	.027*
Sex	.753	.464–1.222	.251			
Grade	1.431	.843–2.430	.185			
Age	1.540	.0954–2.486	.077			
T stage	1.593	1.155–2.198	.005*	1.373	.935–2.017	.106
N stage	1.219	0.983–1.511	.072			
M stage	5.111	2.339–11.168	.000*	3.646	1.228–10.831	.020
TNM stage	1.899	1.297–2.779	.001*	1.216	0.741–1.995	.438

*Statistically significant (*p* < 0.05)

### Inhibition of TRIP13 slows GC development

To evaluate the TRIP13-involved oncogenesis for gastric cancer development, the expression of endogenous *trip13* in gastric cancer cell line was studied, which showed TRIP13 being overexpressed in the five tested gastric cancer cell lines (Fig. 2A). MTT assays demonstrated that TRIP13 knockdown tuned down gastric cancer cell proliferation (Fig. 2B; *P* < 0.05). Additionally, the colony numbers of the group with TRIP13 inhibition were significantly decreased (Fig. 2C; *P* < 0.05). Flow cytometry experiments showed increased apoptosis in the TRIP13 inhibition groups (Fig. 2D; *P* < 0.05). Three replicates were performed in each group in the in vitro experiment above. A xenograft model showed that subcutaneous neoplasms that developed from MGC-803 cells transfected with LVpGCSIL-shTRIP13 were smaller than those from psc3741-shCtrl-transfected MGC-803 cells (Fig. 2E). Ten replicates were performed in each group in the in vivo experiment above. Taken together, these data demonstrate that TRIP13 has carcinogenic potential in the development of GC.

### Microarray analysis of TRIP13 expression in GC

**Fig. 3:**
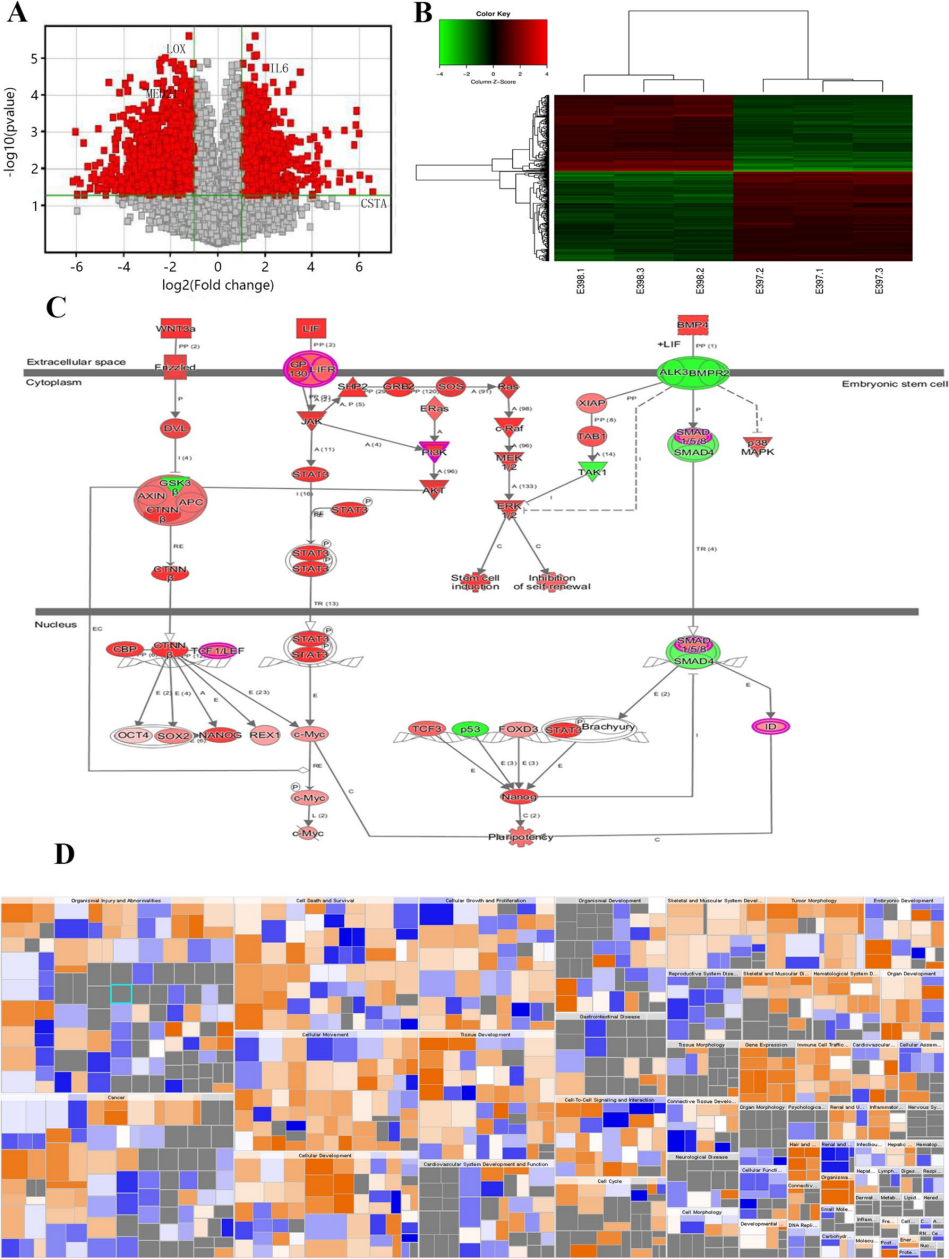
Expression changes of genes and pathways following knockdown of TRIP13. **A** The volcano map shows the distribution of the expressions of different genes after TRIP13 knockdown. The red part is the gene distribution with differential expression greater than 1.5 times. **B** The heat map exhibits the aggregation of all samples and different genes at the expression level. Red indicates the signal value of the relatively upregulated gene; green indicates the signal value of the relatively downregulated gene; black indicates the signal value of the gene with moderate geted ne expression, and gray indicates the signal value of the undetected gene. **C** The experimental data predicted the signal path. **D** Enrichment of differential genes in disease and functional classification

Based on the findings from the above studies, we used a whole human genome expression array (Affymetrix Inc., Santa Clara, CA, USA) to detect genome-wide RNA expression in GC cells with or without TRIP13 knockdown. Three replicates were performed in each group in the in vitro experiment. With good coincidence degree of the obtained signal value distribution curve (Fig. S1A–C), the microarray showed that knockdown of TRIP13 expression could upregulate 271 genes and downregulate 322 genes, which are shown by a volcano plot and heat map (Fig. 3A, B). Enrichment analysis done by ingenuity pathway analysis (IPA) software identified possibly involved pathways (Fig. 3C, D). Through *p*-value and *z*-score calculated by IPA, these 593 differentially expressed genes were found to either activate or inhibit 10 pathways (Table 2), revealing the possible functions of TRIP13 in cell growth, proliferation, death, and survival (Fig. 3D, Table S1). Among these 10 pathways, both JAK/STAT and NF-κB-dependent signaling pathways are classical carcinogenesis-related pathways.

**Table 2:** Ten pathways related to TRIP13

Pathway	-log(*p*-value)	*z*-score	Molecules
Agrin interactions at neuromuscular junction	3.59	1.89	NRG1, GABPB1, JUN, ACTA2, CDC42, UTRN, NRG4, ACTC1
Acute phase response signaling	2.08	1.667	PIK3R3, IL6ST, HMOX1, TCF4, JUN, SOCS6, SOCS2, IL6, AGT, TNFRSF11B
Role of IL-17F in allergic	3.2	1.663	CXCL8, CCL2, RPS6KA5, IL6, CREB5, ATF2
JAK/STAT signaling	1.52	1.342	PIK3R3, JUN, SOCS6, SOCS2, IL6
TREM1 signaling	1.45	1.342	CXCL8, CXCL3, CCL2, CD83, IL6
eNOS signaling	1.24	− 1.414	PRKACB, PIK3R3, AQP3, CASP3, HSPA1A/HSPA1B, HSPA6, HSPA5
Mouse embryonic stem cell	2.66	− 1.633	PIK3R3, IL6ST, LIFR, ID1, TCF4, ID2, SMAD5, ID3
Glioma invasiveness signaling	3.36	− 1.89	PIK3R3, RHOV, TIMP4, RND3, ITGAV, PLAUR, PLAU
NF-κB signaling	0.621	− 2.121	PRKACB, PIK3R3, TNFAIP3, EGF, BTRC, TNFRSF11B
Dendritic cell maturation	1.17	− 2.449	PIK3R3, LEPR, IL15, CD83, IL6, CREB5, TNFRSF11B, ATF2

### Identification of TRIP13-involved pathways

**Fig. 4:**
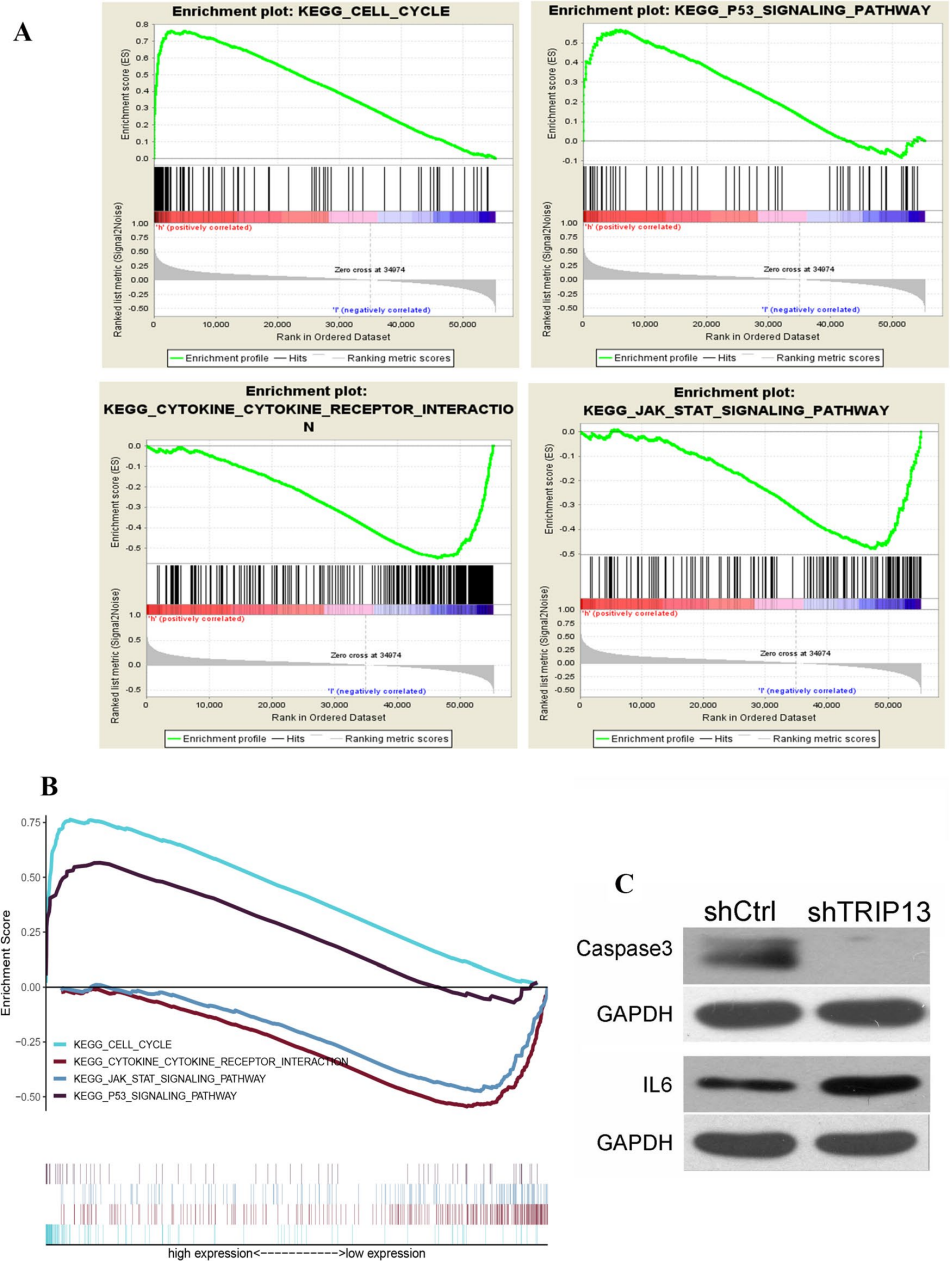
Enrichment plots from GSEA. **A** GSEA results showing differential enrichment of genes related to the cell cycle, p53-dependent pathways, cytokine and receptor binding, and JAK/STAT signaling cascade in GC cases with high TRIP13 expression. **B** Integration of tumor-related pathways. **C** TRIP13 regulation of downstream genes

Both JAK/STAT and NF-κB signaling cascades dictate inflammation-induced carcinogenesis (Khanna et al. 2015; Sokolova and Naumann 2017), activating many biological factors in tandem with exerting their corresponding biological effects. To identify the signaling pathway that regulates TRIP13-dependent carcinogenesis, we proceeded with GSEA to contrast the dataset with either under or overexpression of TRIP13, exhibiting significant differences (FDR < 0.05, *P* < 0.05) in these datasets. Genes involved in the cell cycle, p53-dependent tumor suppression, cytokine and receptor binding, and JAK/STAT signaling pathway were differentially enriched with the expression of TRIP13 (Table 3, Fig. 4A, B). We conclude that the JAK/STAT and p53 pathways may be related to differential TRIP13 expression. Based on the scoring of each protein in the pathway (Tables S2 and S3), corresponding downstream proteins in the pathway were detected and the western blots were quantified. TRIP13 significantly upregulated and downregulated the expression of IL-6 and caspase-3, respectively (Fig. 4C).

**Table 3:** Differences in TRIP13 expression related to different gene pathways in cancer

Gene set name	NES	NOM *p*-val	FDR *q*-val
Cell cycle	2.31	0.00	0.00
p53 signaling pathway	1.87	0.008	0.017
Cytokine receptor interaction	− 2.08	0.004	0.017
JAK/STAT signaling pathway	− 1.83	0.006	0.041

## Discussion

TRIP13 overexpression promotes tumor development, progression, migration, and invasion, underscoring its oncogenic role. Although previous studies have suggested that the overexpression of TRIP13 carries the prognostic potential to predict the development of gastric cancer, there have been very few studies on its mechanism (Dazhi et al. 2017). This study is the first one to unveil TRIP13 as a potential targeted gene for inhibiting the development of GC.

TRIP13 as an AAA + ATPase superfamily member functions in various cellular activities (Hanson and Whiteheart 2005), such as meiotic recombination and DNA repair (Li et al. 2011; Li and Schimenti 2007; Roig et al. 2010). In addition, TRIP13 also plays roles in tumor progression (Banerjee et al. 2014; Grigoletto et al. 2011) and the development of chemotherapeutic resistance. Several studies have discussed the candidacy of TRIP13 in the carcinogenesis of other neoplasms (Banerjee et al. 2014; Larkin et al. 2012; Sheng et al. 2018), and Lu and colleagues (Lu et al. 2019a, b) observed the elevation of TRIP13 expression in gastric adenocarcinoma. In our study, bioinformatics analysis of the TCGA database revealed that TRIP13 has been overexpressed in the cancer cells from the stomach compared with the normal gastric cells, confirming that TRIP13 is an oncogene for gastric cancer. Since gastric cancer possesses various malignant types, the levels of TRIP13 expression were studied in gastric tissues from 99 gastric cancer patients and established that TRIP13 expression status was significantly subjective to tumor-node-metastasis (TNM) staging and poor survival, suggesting that TRIP13 can be considered as an independent prognostic indicator for gastric cancer.

To confirm that TRIP13 possesses oncogenic potential, we selected five GC cell lines and found abundant TRIP13 expression in all five cell lines by qPCR. Then, we sub-selected AGS and MGC-803 from the five cell lines and knocked down TRIP13 expression. The downregulation of TRIP13 promoted apoptosis and inhibited tumor growth. The oncogenic role of TRIP13 has been established in the development of other cancers. Wang et al. (2020) reported in their study that the TRIP13 inhibitor did cause multiple myeloma cell death by downplaying the NF-κB signaling pathway (Wang et al. 2020). Lu et al. (2019a, b) verified that TRIP13 elicited oncogenic function in bladder cancer through knocking out spindle assembly-dependent checkpoint signaling. With the aim of exploring TRIP13 biological function and elucidating the related mechanisms in GC, we knocked down the expression of TRIP13, consequently identifying 271 upregulated genes and 322 downregulated genes through mRNA expression profile microarray, and mapped out 10 TRIP13-related signaling pathways by IPA analysis. GSEA further identified TRIP13-dependent JAK/STAT and NF-κB signaling cascade as two key pathways in the carcinogenesis of GC.

In the stomach, chronic inflammation from *Helicobacter pylori* infection accounts for about 90% of non-cardia GC cases (Plummer et al. 2015). The underlying carcinogenesis related to *H. pylori* is a multistep process. Early DNA damage to gastric mucosa leads to an increase in NF-κB expression, which subsequently promotes the release of inflammatory mediators in the cancer tissue compared with adjacent normal mucosa of stomach cancer (Yin et al. 2013). However, the results from western blotting in our study showed that IL-6 expression increased when TRIP13 was knocked down, indicating that other pathways may affect the expression of IL-6.

GSEA was attempted to compare the dataset with under- and overexpression of TRIP13, finding that the JAK/STAT and p53 pathways may regulate differential TRIP13 expression. The JAK/STAT signaling cascade functions in a range of intracellular processes. The JAK/STAT cascade was originally identified in the context of interferon-α (IFN-α), IFN-γ, and interleukin-6 (IL-6)-mediated signaling activities (Khanna et al. 2015). When TRIP13 was knocked down in GC cells, the JAK/STAT signaling cascade was activated to upregulate the expression of IL-6. The carcinogenic role of the p53 signaling pathway has been shown in numerous studies, demonstrating that the loss of p53 function promotes the development of about 50% of human tumors (Lane and Levine 2010). Previous studies have shown that p53 as the caretaker gene can halt cell cycle and induce apoptosis in response to acute DNA damage (Bieging et al. 2014; Lane and Levine 2010). To determine whether TRIP13 regulates the p53 signaling pathway, we selected caspase-3 as a p53 signaling-related protein and further confirmed the positive effect of p53 signaling after TRIP13 knockdown.

## Conclusions

In conclusion, TRIP13 participates in the carcinogenesis of stomach cancer, and its overexpression in the cancerous tissues from gastric malignancy dovetail with advanced stage and survival. Moreover, TRIP13 functions as an upstream regulator of more than 593 downstream genes involving 10 signaling pathways, including the JAK/STAT and p53 signaling pathways, which play critical roles in developing various malignancies. These results suggest that TRIP13 is not only a prognostic marker for GC but also a candidate for future targeted therapy.

## Supplementary Information

Below is the link to the electronic supplementary material.

Supplementary file1 (DOC 1385 KB)

## Data Availability

The dataset used and/or analyzed in this study is available from the corresponding author on reasonable request.
