# Supplementary material for: TRIP 13-dependent pathways promote the development of gastric cancer
Source: Funct Integr Genomics. 2023 Jul 11;23(3):232. doi: 10.1007/s10142-023-01160-7 (PMC10335954; doi:10.1007/s10142-023-01160-7)
Supplement: Supplementary file 1 — Supplementary file1 (DOC 1385 KB) [file 10142_2023_1160_MOESM1_ESM.doc]

Supplementary Material

## Supplementary Figure


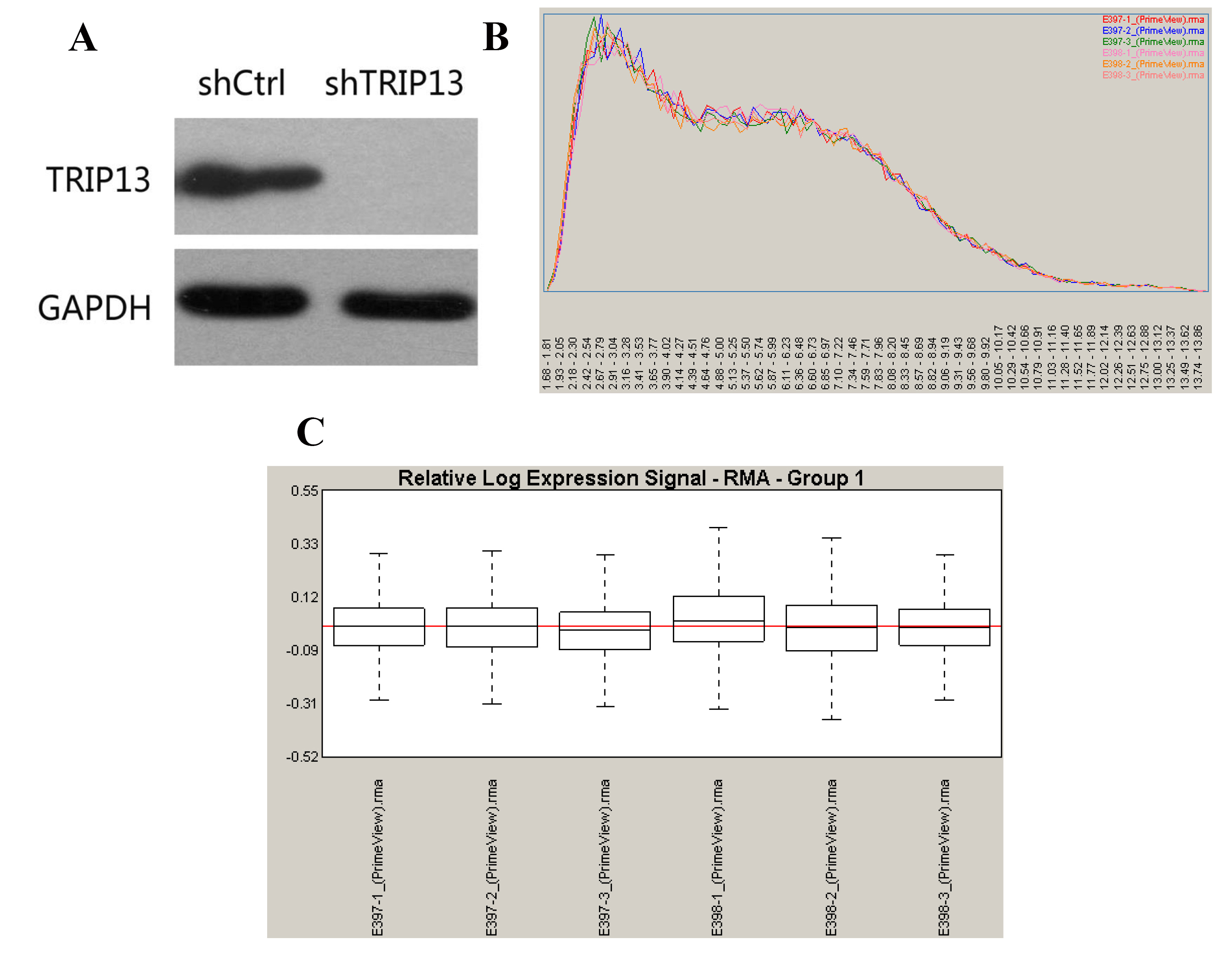


**Figure S1 Status of lentivirus transfection and corresponding data of gene chips**

**A.** Western blot detected TRIP13 expression after lentivirus transfection. **B.** The signal value distribution graph shows the distribution statistics of all the chip probes. **C.** The relative logarithm representation box plot shows the distribution of the relative logarithm representation of all chips.

**Table S1** Enrichment of differential genes in disease and functional classification

| **Categories** | **Diseases or Functions Annotation** | **p-Value** | **Activation z-score** |
| --- | --- | --- | --- |
| Cell Death and Survival | apoptosis | 1.9E-13 | 1.122 |
| Cellular Growth and Proliferation | Proliferation of cells | 3.48E-13 | -1.551 |
| Cell Death and Survival | cell death | 4.04E-13 | 0.168 |
| Cell Death and Survival | necrosis | 9.13E-13 | 0.385 |
| Cancer, Organismal Injury and Abnormalities | growth of tumor | 2.81E-12 | -0.103 |
| Cancer, Organismal Injury and Abnormalities | cancer | 4.35E-12 | 0.729 |
| Cellular Movement | cell movement | 9.84E-12 | -1.134 |
| Cancer, Organismal Injury and Abnormalities | malignant solid tumor | 1.03E-11 | 0.306 |
| Cellular Movement, Skeletal and Muscular System Development and Function | cell movement of smooth muscle cells | 1.28E-11 | 0.391 |
| Cell Death and Survival | necrosis of epithelial tissue | 2.82E-11 | -0.912 |

**Table S2** Scores of genes in the P53 pathway in GSEA

| **PROBE** | **RANK IN GENE LIST** | **RANK METRIC SCORE** | **RUNNING ES** | **CORE ENRICHMENT** |
| --- | --- | --- | --- | --- |
| CCNB2 | 6 | 0.679573596 | 0.0531193 | Yes |
| CCNB1 | 33 | 0.61262095 | 0.1006322 | Yes |
| GTSE1 | 55 | 0.571763992 | 0.14503552 | Yes |
| CHEK1 | 60 | 0.56750834 | 0.18941349 | Yes |
| CDK1 | 70 | 0.558125317 | 0.23296593 | Yes |
| CHEK2 | 78 | 0.551852226 | 0.27606326 | Yes |
| RRM2 | 157 | 0.500059962 | 0.31381777 | Yes |
| CDK2 | 363 | 0.419902682 | 0.34299332 | Yes |
| CCNE2 | 437 | 0.405133277 | 0.37340325 | Yes |
| CYCS | 451 | 0.401640743 | 0.4046265 | Yes |
| CDKN2A | 1070 | 0.324302465 | 0.4188326 | Yes |
| BID | 1247 | 0.308868349 | 0.43983668 | Yes |
| CCNE1 | 1451 | 0.294458926 | 0.459223 | Yes |
| EI24 | 1608 | 0.283860266 | 0.47863057 | Yes |
| CDK4 | 1858 | 0.269050568 | 0.4951935 | Yes |
| CCNB3 | 2286 | 0.25067547 | 0.5070927 | Yes |
| CASP3 | 2317 | 0.250017643 | 0.52613205 | Yes |
| PMAIP1 | 2985 | 0.22534579 | 0.5316997 | Yes |
| RCHY1 | 3114 | 0.221308678 | 0.54671514 | Yes |
| TP73 | 3790 | 0.202775896 | 0.55037004 | Yes |
| PPM1D | 4453 | 0.188538581 | 0.55314535 | Yes |
| CCND1 | 4924 | 0.179521725 | 0.55869246 | Yes |
| STEAP3 | 5310 | 0.172708437 | 0.5652457 | Yes |
| PERP | 5961 | 0.16230385 | 0.5661835 | Yes |

**Table S3** Scores of genes in the JAK/Stat pathway in GSEA

| **PROBE** | **RANK IN GENE LIST** | **RANK METRIC SCORE** | **RUNNING ES** | **CORE ENRICHMENT** |
| --- | --- | --- | --- | --- |
| CLCF1 | 47834 | -0.1027 | -0.47416 | Yes |
| IFNAR1 | 47863 | -0.10302 | -0.46911 | Yes |
| PIM1 | 48035 | -0.10616 | -0.46648 | Yes |
| IL2RA | 48142 | -0.10774 | -0.46259 | Yes |
| SPRED1 | 48173 | -0.1082 | -0.45729 | Yes |
| IL20RB | 48196 | -0.10857 | -0.45183 | Yes |
| SPRY3 | 49060 | -0.1229 | -0.46086 | Yes |
| TPO | 49075 | -0.12311 | -0.45447 | Yes |
| IL6R | 49592 | -0.13215 | -0.4567 | Yes |
| IL21 | 49601 | -0.13237 | -0.4497 | Yes |
| LEP | 49891 | -0.13845 | -0.44747 | Yes |
| PIK3CG | 49978 | -0.1404 | -0.44145 | Yes |
| CTF1 | 50119 | -0.14314 | -0.43627 | Yes |
| IL2 | 50150 | -0.14374 | -0.42905 | Yes |
| IL21R | 50265 | -0.14615 | -0.42323 | Yes |
| SOCS2 | 50397 | -0.14934 | -0.41755 | Yes |
| OSMR | 50436 | -0.15031 | -0.41013 | Yes |
| CBLB | 50711 | -0.15604 | -0.40668 | Yes |
| IL5 | 50848 | -0.15951 | -0.40054 | Yes |
| PIK3R1 | 51056 | -0.16458 | -0.39541 | Yes |
| SOCS3 | 51090 | -0.16573 | -0.38706 | Yes |
| SOS2 | 51104 | -0.16618 | -0.37833 | Yes |
| SOCS5 | 51386 | -0.17426 | -0.37402 | Yes |
| STAT3 | 51432 | -0.17544 | -0.36537 | Yes |
| STAT2 | 51506 | -0.1773 | -0.35713 | Yes |
| IL15 | 51540 | -0.1783 | -0.3481 | Yes |
| IL2RG | 51630 | -0.1808 | -0.33996 | Yes |
| CSF2RA | 51675 | -0.18201 | -0.33094 | Yes |
| IL10RA | 51685 | -0.18215 | -0.32127 | Yes |
| CREBBP | 51755 | -0.18426 | -0.31258 | Yes |
| IL5RA | 52343 | -0.20412 | -0.31221 | Yes |
| IL12RB1 | 52365 | -0.20502 | -0.30153 | Yes |
| IL11RA | 52391 | -0.20576 | -0.29087 | Yes |
| IL2RB | 52447 | -0.20779 | -0.28066 | Yes |
| IL12B | 52659 | -0.21683 | -0.27278 | Yes |
| STAT5A | 52948 | -0.22835 | -0.26568 | Yes |
| IFNAR2 | 52970 | -0.22915 | -0.2537 | Yes |
| JAK3 | 53076 | -0.23319 | -0.24302 | Yes |
| CSF2RB | 53138 | -0.23547 | -0.23142 | Yes |
| JAK1 | 53146 | -0.23588 | -0.21881 | Yes |
| IL9R | 53403 | -0.24783 | -0.21008 | Yes |
| STAT5B | 53518 | -0.25514 | -0.19838 | Yes |
| SPRY1 | 53575 | -0.25888 | -0.18542 | Yes |
| IL7R | 53606 | -0.26048 | -0.17191 | Yes |
| LIFR | 53689 | -0.26529 | -0.15908 | Yes |
| STAT4 | 53726 | -0.26768 | -0.14529 | Yes |
| PIK3CD | 54060 | -0.28709 | -0.13583 | Yes |
| PIK3R5 | 54191 | -0.29577 | -0.12223 | Yes |
| TSLP | 54458 | -0.31638 | -0.10998 | Yes |
| IL4R | 54471 | -0.31852 | -0.09301 | Yes |
| LEPR | 54775 | -0.35362 | -0.07942 | Yes |
| GHR | 54924 | -0.37303 | -0.06197 | Yes |
| AKT3 | 55080 | -0.40314 | -0.04303 | Yes |
| IL3RA | 55097 | -0.40937 | -0.02122 | Yes |
| IL6ST | 55204 | -0.45006 | 0.001143 | Yes |
